# Comparative Analyses of mTOR/Akt and Muscle Atrophy-Related Signaling in Aged Respiratory and Gastrocnemius Muscles

**DOI:** 10.3390/ijms21082862

**Published:** 2020-04-20

**Authors:** Kun Woo Kim, Hye-Jeong Cho, Sana Abdul Khaliq, Kuk Hui Son, Mee-Sup Yoon

**Affiliations:** 1 Department of Thoracic and Cardiovascular Surgery, Gachon University Gil Medical Center, College of Medicine, Gachon University, Incheon 21565, Korea; isee03@gilhospital.com; 2 Department of Molecular Medicine, School of Medicine, Gachon University, Incheon 21999, Korea; hyejeong.cho77@gmail.com (H.-J.C.); sanaakhaliq1@gmail.com (S.A.K.); 3 Lee Gil Ya Cancer and Diabetes Institute, Gachon University, Incheon 21999, Korea; 4 Department of Health Sciences and Technology, GAIHST, Gachon University, Incheon 21999, Korea

**Keywords:** sarcopenia, diaphragm, gastrocnemii, intercostal muscles, muscle atrophy, autophagy

## Abstract

Sarcopenia is the degenerative loss of skeletal muscle mass and function associated with aging and occurs in the absence of any underlying disease or condition. A comparison of the age-related molecular signaling signatures of different muscles has not previously been reported. In this study, we compared the age-related molecular signaling signatures of the intercostal muscles, the diaphragm, and the gastrocnemii using 6-month and 20-month-old rats. The phosphorylation of Akt, ribosomal S6, and Forkhead box protein O1 (FoxO1) in diaphragms significantly increased with age, but remained unchanged in the intercostal and gastrocnemius muscles. In addition, ubiquitin-proteasome degradation, characterized by the levels of MuRF1 and Atrogin-1, did not change with age in all rat muscles. Interestingly, an increase in LC3BII and p62 levels marked substantial blockage of autophagy in aged gastrocnemii but not in aged respiratory muscles. These changes in LC3BII and p62 levels were also associated with a decrease in markers of mitochondrial quality control. Therefore, our results suggest that the age-related signaling events in respiratory muscles differ from those in the gastrocnemii, most likely to preserve the vital functions played by the respiratory muscles.

## 1. Introduction

The skeletal muscle is a major metabolic organ and makes up 40% of our body mass [1]. Sarcopenia is defined as the age-associated degenerative loss of muscle mass, quality, and strength [2,3]. Sarcopenia is a gradual process that increases the risk of disability, frailty, and death. Its progression is dependent on exercise levels, nutrition status, and mobility [4]. Sarcopenia is attributed to an imbalance between protein synthesis and degradation [5]. Two major proteolytic pathways in the muscles, ubiquitin-related protein degradation and autophagy, have been linked to protein homeostasis imbalance. However, it has been reported that the decrease in muscle strength with age is not directly related to the loss of muscle mass [6], which suggests that there are distinct regulatory mechanisms maintaining muscle mass and strength.

Phosphatidylinositol 3-kinase (PI3K)-Akt-mammalian target of rapamycin (mTOR) plays a key role in determining whether a muscle grows or atrophies [7]. PI3K-Akt primarily triggers muscle mass growth by activating protein synthesis through mTOR complex 1 (mTORC1) signaling. mTOR, a master regulator of cell growth, is a serine/threonine kinase that is well conserved across species ranging from yeast to mammals [8]. mTORC1 directly phosphorylates the eukaryotic translation initiation factor 4E (eIF4E)-binding protein 1 (4E-BP1) and S6 kinase 1 (S6K1). The phosphorylation of 4E-BP1 helps to initiate cap-dependent translation by impeding its binding to eIF4E and the subsequent formation of the eIF4F complex [9]. The phosphorylation of S6K1/ribosomal S6 enhances mRNA biogenesis along with translational initiation and elongation [9]. In addition, activation of PI3K-Akt signaling enhances protein synthesis via the repression of Forkhead box protein O (FoxO) transcription factors [7]. FoxO mediates the expression of the atrogene program, which causes substantial proteolysis in muscles [10]. Hence, the loss of muscle does not appear to simply be the opposite of muscle hypertrophy.

Autophagy is an important physiological process for growth maintenance and tissue homeostasis in multicellular organisms. Autophagosomes are double-membrane lines vesicles that maintain cellular homeostasis by sequestering cytosolic substrates, fusing to lysosomes to form autophagolysosomes, and subsequently degrading damaged/dysfunctional proteins and organelles [11]. Therefore, autophagy is a critical process involved in protecting cells against cellular stress and aging-associated damage.

Respiratory muscles are essential for the expansion and compression of the chest wall [12]. Although the diaphragm is the main respiratory muscle, other muscles are engaged more under high metabolic demand [13]. The intercostal muscles are one of the most important respiratory muscles as they operate the movement of the rib cage [13]. Healthy individuals do not present difficulty in alveolar ventilation at rest or during low-intensity exercise. In contrast, susceptibility to clinically observed respiratory complications, such as pneumonia and respiratory infection, is higher among the elderly [14,15], in whom respiratory muscle sarcopenia is likely to be present [16,17,18,19,20]. The diaphragm muscles decrease in size and function with age [21]. In addition, a significant decrease in the cross-sectional area of intercostal muscles has been reported in patients with chronic obstructive pulmonary disease (COPD) [22]. Although the age-related loss of respiratory muscle function and mass has been reported, there have been no studies comparing the specific molecular signaling signatures of protein homeostasis and muscle function in different muscles.

It remains unknown whether similar signaling maintains aged respiratory muscles and aged extremity muscles. In the present study, we analyzed and compared the molecular signaling signatures of respiratory muscles and gastrocnemii. We found that the activities of mTORC1 and Akt, the well-known signaling molecules of protein synthesis, remained relatively unchanged. Similarly, the levels of MuRF1 and Atrogin-1, which are related to ubiquitin-dependent protein degradation, also remained relatively unchanged. The defects in autophagy and mitochondrial quality control were significant only in the gastrocnemii. Our findings suggest that, compared to the gastrocnemii, respiratory muscles tend to resist age-related cellular damage.

## 2. Results

### 2.1. Comparison of the Activities of mTORC1 and Akt in Respiratory Muscles and Gastrocnemii as a Function of Age

**Figure 1:**
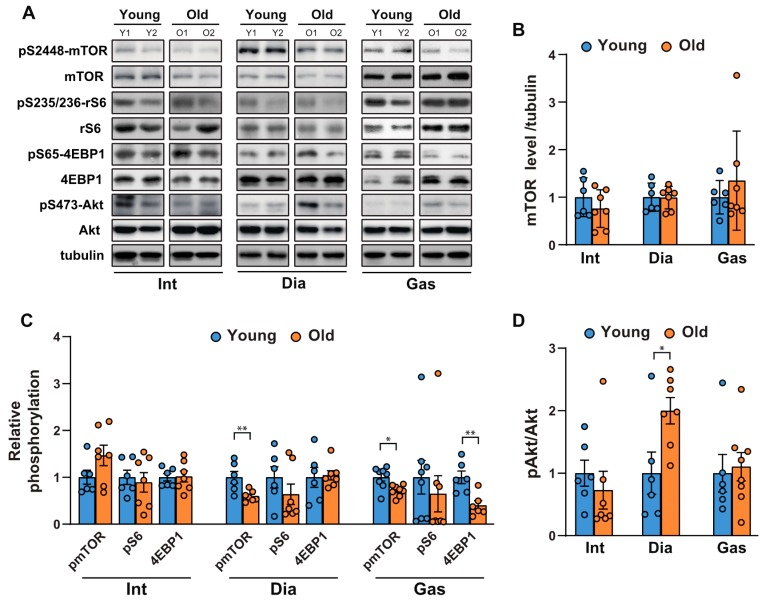
Comparison of the age-related changes in activities of mTORC1 and Akt in the respiratory muscles and gastrocnemii. (**A**) Each muscle was lysed, and Western blot analysis was performed (*n* = 6). (**B**–**D**) The relative intensities of the bands were quantified using ImageJ analysis software (*n* = 6). (**B**) Data are presented for mTOR compared to tubulin, (**C**) pSer2448-mTOR compared to mTOR, pSer235/236-ribosomal S6 compared to ribosomal S6, pSer65-4EBP1 compared to 4EBP1, and (**D**) pS473-Akt compared to Akt. The data are shown as the mean ± standard deviation. Statistical analysis was performed using unpaired Student’s t-test. * *p* < 0.05; ** *p* < 0.01; 6-month-old versus 20-month-old rat muscles. Abbreviations: diaphragm muscle (Dia); gastrocnemius muscle (Gas); intercostal muscle (Int); 6-month-old rat (Young); 20-month-old rat (Old).

To investigate the age-related changes in protein synthesis and degradation in muscles, we first examined the activities of mTORC1 and Akt in respiratory muscles, including the intercostal and diaphragm muscles, and in an extremity muscle, the gastrocnemius, in 6-month-old and 20-month-old rats. mTORC1 regulates protein synthesis by phosphorylating S6K1 and 4EBP1, which subsequently regulate the translational machinery. As shown in Figure 1A,B, mTOR protein levels remained unchanged; however, mTORC1 activity decreased mildly in the gastrocnemii of 20-month-old versus those of 6-month-old rats (Figure 1A,C). This was evidenced by the reduction in the phosphorylation of mTOR at Ser 2448, ribosomal S6 at Ser 235/236, and 4EBP1, a direct mTORC1 substrate, at Ser 65 (Figure 1A,C). On the contrary, mTORC1 activity in the respiratory muscles was inconsistent; phosphorylation of mTOR at Ser 2448 decreased and remained unchanged in the diaphragm and intercostal muscles, respectively (Figure 1A,C). The phosphorylation of ribosomal S6 and 4EBP1 did not change significantly in the respiratory muscles (Figure 1A,C). On the other hand, Akt phosphorylation at Ser 473 significantly increased in the diaphragm (Figure 1A,D), suggesting that a decrease in mTOR phosphorylation led to the removal of the negative feedback regulation on insulin receptor substrate-1 (IRS-1) and subsequently activated Akt [23]. However, no significant change in Akt phosphorylation in the intercostal and gastrocnemius muscles of old rats was found (Figure 1A,D). Hence, aging appears to result in a mTORC1 signaling decline in the diaphragm muscles and gastrocnemii, while significantly increasing Akt phosphorylation in the diaphragm muscles.

### 2.2. Comparison of the Age-related Changes in Activities of FoxO1, mRNA Levels of Klf15, and Ubiquitin-related Proteinases Between the Respiratory Muscles and Gastrocnemii

**Figure 2:**
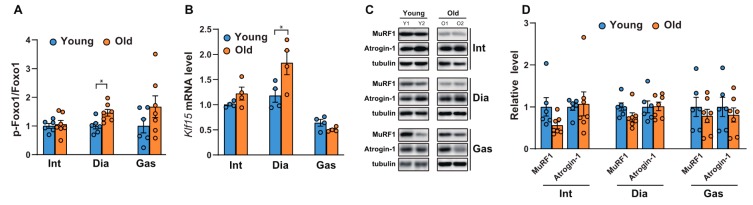
Comparison of the phosphorylation of FoxO1 and the expression levels of *Klf15* and ubiquitin-related proteinases between the respiratory muscles and gastrocnemii with age. (**A**,**C**) Each muscle was lysed and analyzed by Western blotting (*n* = 6). The relative intensities of the bands were quantified using ImageJ analysis software (*n* = 6). Data are displayed for pSer256-FoxO1 compared to those for FoxO1. (**B**) The muscles were lysed and subjected to RT-qPCR analysis (*n* = 6). Rat glyceraldehyde 3-phosphate dehydrogenase (*Gapdh)* was used to normalize gene expression. (**D**) The relative intensities of the bands were quantified using ImageJ analysis software (*n* = 6). MuRF1 and Atrogin-1 levels are both shown in comparison with tubulin levels. The data are shown as the mean ± standard deviation. Statistical analysis was performed using unpaired Student’s t-test. * *p* <0.05; 6-month-old versus 20-month-old rat muscles. Abbreviations: diaphragm muscle (Dia); gastrocnemius muscle (Gas); intercostal muscle (Int); 6-month-old rat (Young); 20-month-old rat (Old).

We next analyzed the phosphorylation of FoxO, since we observed differential activation of Akt in the diaphragm muscles. FoxOs are well-known to be the downstream targets of Akt and act as transcription factors to regulate atrophy-related genes in muscles, such as MuRF-1 and Atrogin1 [24]. As shown in Figure 2A and Supplementary Figure S1, the phosphorylation of FoxO1 at Ser 256 increased significantly in aged diaphragm muscles but remained unchanged in the intercostal and gastrocnemius muscles. This result was in accordance with the significant increase observed in Akt phosphorylation at Ser 473 in aged diaphragm muscles (Figure 1A,D). On the contrary, the mRNA level of *Klf15*, one of several Kruppel-like, zinc finger transcription factors (KLFs), increased significantly only in aged diaphragm muscles (Figure 2B). Expression of KLF15 is induced by glucocorticoids, resulting in the expression of MuRF1 and Atrogin-1, both E3 ubiquitin ligases in skeletal muscles [25]. These proteins bind to polyubiquitinated proteins to direct them towards subsequent degradation by the 26S proteasome [26]. Accordingly, we examined the protein expression levels of MuRF1 and Atrogin-1. Our results showed that the MuRF1 levels decreased mildly, but not significantly, in all aged respiratory muscles and gastrocnemii, whereas levels of Atrogin-1 remained relatively unchanged (Figure 2C,D). Notably, increases in FoxO1 phosphorylation and *Klf15* expression were not effective at reducing the expression levels of MuRF1 and Atrogin-1, suggesting the possibility of another regulatory signaling pathway, responsible for protein degradation in aged diaphragm muscles. These results suggest that protein degradation by MuRF1 and Atrogin-1 might not be a primary mechanism in aged muscles.

### 2.3. Analysis of the Autophagic Flux between the Respiratory Muscles and Gastrocnemii with Age

**Figure 3:**
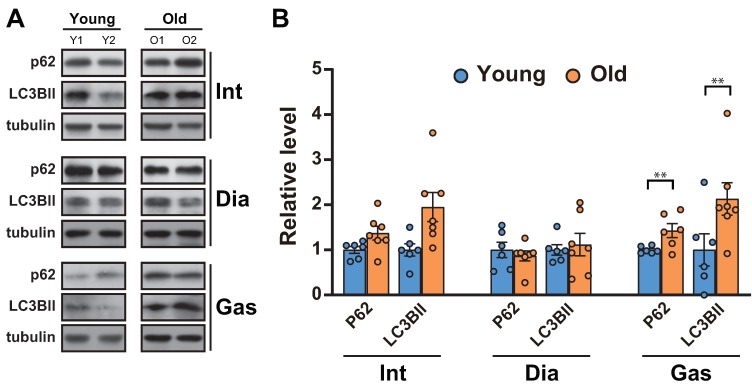
Autophagic flux was blocked in the gastrocnemii of aged rats. (**A**) The intercostal, diaphragm, and gastrocnemius muscles were lysed and subjected to Western blot analysis (*n* = 6). (**B**) The relative intensities of the bands were quantified using ImageJ analysis software (*n* = 6). Data for LC3BII and p62 were compared to levels of tubulin. The data are shown as the mean ± standard deviation. Statistical analysis was performed using unpaired Student’s t-test. ** *p* < 0.01; 6-month-old versus 20-month-old rat muscles. Abbreviations: diaphragm muscle (Dia); gastrocnemius muscle (Gas); intercostal muscle (Int); 6-month-old rat (Young); 20-month-old rat (Old).

As the ubiquitin proteasome-degradation markers, MuRF1 and Atrogin-1, remained unchanged in muscles, we next analyzed autophagic flux, a proteolytic system distinct from ubiquitination in muscles [27]. Autophagy is known to aid in maintaining muscle function by clearing the damaged proteins/organelles [1]. A decrease in autophagic flux, indicated by increases in p62 and LC3BII, occurred in the gastrocnemii (Figure 3A,B), as previously reported [11]. On the other hand, the levels of p62 and LC3BII remained unchanged in 20-month-old diaphragm muscles and increased in 20-month-old intercostal muscles compared to those in 6-month-old muscles, although the change was not significant (Figure 3A,B).

**Figure 4:**
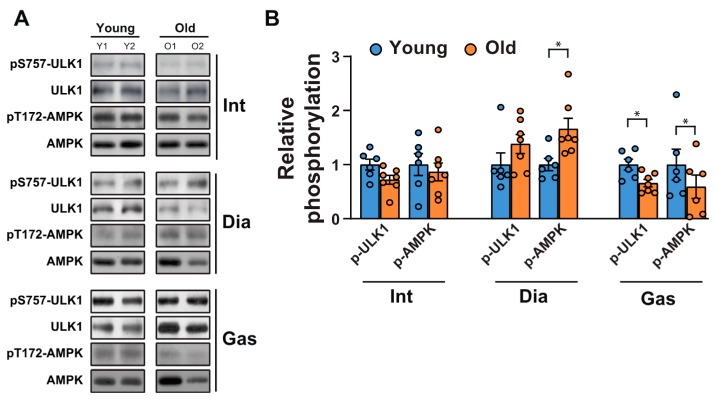
Changes in autophagy initiating signaling between the respiratory muscles and gastrocnemii with age. (**A**) The intercostal, diaphragm, and gastrocnemius muscles were lysed and subjected to Western blot analysis (*n* = 6). (**B**) The relative intensities of the bands were quantified using the ImageJ analysis software (*n* = 6). Data are presented for pSer757-ULK1 compared to those for ULK1 and for pThr172-AMPK compared to those for AMPK. The data are shown as the mean ± standard deviation. Statistical analysis was performed using unpaired Student’s t-test. * *p* < 0.05; 6-month-old versus 20-month-old rat muscles. Abbreviations: diaphragm muscle (Dia); gastrocnemius muscle (Gas); intercostal muscle (Int); 6-month-old rat (Young); 20-month-old rat (Old).

Next, we tested whether a decrease in autophagic flux in the gastrocnemii was due to the initiation signals of autophagy. AMP-activated protein kinase (AMPK) is an energy stress sensor that maintains energy homeostasis [28]. It prompts autophagy by directly phosphorylating Unc-51 like autophagy activating kinase 1 (ULK1), a mammalian autophagy initiating kinase, at Ser 317 and Ser 777 [28]. AMPK phosphorylation at Thr 172, a phosphorylation required for AMPK activation [29], increased significantly in the aged diaphragm muscles but not in the aged intercostal muscles (Figure 4A,B). In contrast, its phosphorylation was reduced in the gastrocnemii of 20-month-old rats compared to those of 6-month-old rats (Figure 4A,B). In addition, ULK 1 phosphorylation at Ser 757, a target site of mTORC1, decreased significantly in aged gastrocnemii (Figure 4A,B), in line with a decrease in mTORC1 activity (Figure 1A,C). ULK1 phosphorylation at Ser 757 by mTOR disrupts the interaction between ULK1 and AMPK and consequently impedes autophagy initiation [28]. ULK1 phosphorylation at Ser 757 in the intercostal muscles did not differ significantly between 6-month-old and 20-month-old rats. In addition, ULK1 phosphorylation was enhanced in 20-month-old diaphragm muscles, but not significantly. These results suggest that a mild decrease in AMPK activation may be sufficient to block autophagic flux in the gastrocnemii of 20-month-old rats despite a decrease in ULK1-inhibitory phosphorylation by mTOR.

### 2.4. Examination of Mitochondrial Quality Control-related Gene mRNA Levels Between Respiratory Muscles and Gastrocnemii with Age

**Figure 5:**
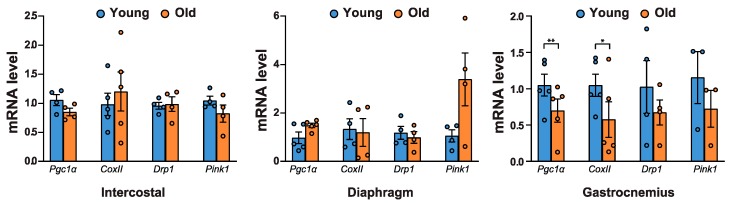
mRNA levels of mitochondrial quality-related genes in aged muscles. Each muscle was lysed and subjected to RT-qPCR analysis (*n* > 4). Rat *18S rRNA* was used to normalize gene expression. The data are shown as the mean ± standard deviation. Statistical analysis was performed using unpaired Student’s t-test. * *p* < 0.05; ** *p* < 0.01; 6-month-old versus 20-month-old rat muscles. Abbreviations: 6-month-old rat (Young); 20-month-old rat (Old).

The blockage of autophagy results in the accumulation of p62 and ubiquitylated proteins, as well as an increase in mitochondrial dysfunction [1]. Hence, we examined whether mitochondrial biogenesis and mitochondrial content changed with age. The mRNA levels of *Pgc1α,* a key factor for mitochondrial biogenesis [30], and *CoxII*, a mitochondrial protein, decreased significantly in aged gastrocnemii (Figure 5). Next, we assessed the mRNA levels of mitochondrial quality control markers. DRP1 is associated with mitochondrial fission and subsequent mitophagy [31]. PINK1 is a marker for damaged mitochondria, targeting their degradation specifically by mitophagy [32]. Both *Drp1* and *Pink1* mRNA levels decreased in aged gastrocnemii (Figure 5). However, neither of these mitochondrial factors changed significantly in intercostal and diaphragm muscles. These results suggest that defective autophagy is associated with a decrease in mitochondrial biogenesis and reduced mitochondrial quality control in aged gastrocnemii.

## 3. Discussion

The regulation of respiratory muscles is assumed to be different from that of extremity muscles. However, a comparison of their signaling signatures has not previously been reported. In the present study, we found that autophagy is impaired in aged gastrocnemii, resulting in a defect in mitochondrial quality control (Table 1). In contrast, autophagy did not appear to be compromised in respiratory muscles (Table 1). We found that the defect in autophagy in aged gastrocnemii causes differential muscle function regulation compared to that in respiratory muscles (Table 1).

Rats are widely used as a model organism and play an important role in the understanding of human biology and disease. In line with this, a recent research article, using genome-scale network reconstruction (GENRES), showed that rats and humans share the majority of their biochemical capabilities at the genome level [33]. Despite that, several physiological and metabolic genes show differential expression between humans and rats, leading to physiological differences between the two species. Analysis of the core proteome of human and rat pancreatic beta cells using label-free LC-MS/MS revealed that human beta cells differ from rat beta cells in glucose sensing enzymes, heat shock proteins, and radical scavenging systems [34]. Also, plasma membrane fractions of choroid plexuses from Wistar rats showed a differential expression pattern of blood-cerebrospinal fluid barrier transporters than humans [35]. Because these reports indicate the limitations of rats as a model to simulate human physiology, caution should be exercised when applying observations from rat-based studies to human subjects.

In the present study, we analyzed the muscles of 20-month-old and 6-month-old rats, the age ranges used in prior studies (Supplementary Table S1). Further, a recent paper showed that gene expression signatures in muscles from 21 month-old rats were similar to those from 24- and 27-month-old rats [36], suggesting that 20 month-old rats are representative of old-aged muscle. Our observations are consistent with previous reports regarding defects in autophagy and mitochondrial quality control in aged gastrocnemii [1,11], indicating that the age range of 6 month- and 20 month-old rats is acceptable to study the aged muscle.

Two important regulations associated with muscle atrophy are ubiquitin-related protein degradation and autophagy [27]. Our results suggest that there are no significant changes in Atrogin-1 and MuRF1, primary well-known E3 ligases in muscles (Figure 2C,D), or autophagic flux in the respiratory muscles (Figure 3A,B), whereas a decrease in autophagy was observed in the gastrocnemius muscles (Figure 3A,B). Based on our results, we propose that the atrophy-induced signaling in the respiratory muscles might not be enhanced compared to one in the gastrocnemius. Autophagy is a prominent degradation process for recycling damaged or aged organelles and maintaining proper muscle function [5]. Notably, the prolonged existence of dysfunctional organelles in muscles is likely to induce catabolic pathways, resulting in muscle atrophy and weakness [1]. Autophagy, acting in concert with ubiquitin-related protein degradation, has been assumed to be responsible for the degradation of proteins in the process of sarcopenia. However, the loss of muscle strength in aged muscles does not directly correspond to the loss of muscle mass [6], suggesting that the loss of muscle strength does not stem entirely from muscle atrophy, which is the primary feature of sarcopenia. Additionally, defective protein degradation and the subsequent accumulation of damaged proteins and dysfunctional organelles results in the loss of muscle mass in aged muscles. This is in line with the muscle-specific knockout of the key autophagy genes Atg5 and Atg7, which results in muscles that are defective in autophagy and accumulate p62–poly-ubiquitin protein inclusions, and have abnormal mitochondria and sarcomeric deformities [1,37]. These findings support the requirement of autophagy in muscle function maintenance by clearing damaged proteins/organelles and ubiquitylated proteins, which are autophagic substrates [38]. The accumulation of p62, as well as ubiquitylated proteins, causes cytotoxicity, resulting in the degeneration or dysfunction of muscles; this suggests that autophagy plays a homeostatic role in post-mitotic differentiated cells [39]. Moreover, autophagy is closely connected with mitochondria quality control, which is orchestrated through mitochondrial turnover [1]. This process repairs and/or removes damaged mitochondria, aiding the subsequent synthesis of new mitochondria [11]. Our results suggest that the ablation of autophagy in aged gastrocnemii may lead to a defect in mitochondrial quality control and, consequently, the dysfunction of aged gastrocnemii (Figure 5). Notably, an autophagic defect was not observed in aged respiratory muscles, as indicated by the levels of p62 and LC3BII (Figure 3A,B). These results indicate that autophagy may be preserved in aged respiratory muscles by respiratory-specific regulation, which warrants further investigation.

The loss of muscle mass largely stems from disturbed signaling pathways involved in the maintenance of the anabolism/catabolism balance of muscle protein, shifting toward a more prominent catabolic state [40]. It is well known that the regulation of proteolysis in muscles is more complex and depends on the transcription of genes regulating the proteolytic mechanism [41]. The expression of the regulators of protein degradation/ubiquitination should be analyzed to provide a measurement of protein degradation/ubiquitination [41]. In the present study, we focused on two important regulators of protein degradation, in muscles; MuRF-1 and Atrogin1. Hence, analyzing the expression levels of MuRF1 and Atrogin-1 in different muscles can assess changes in protein degradation/ubiquitination activity. In the present study, we observed that the levels of MuRF1 and Atrogin-1 remained relatively unchanged (Figure 2C,D). Although they were mild, significant increases in *Klf15* expression were observed in the diaphragm muscles, whereas no significant changes in MuRF1 and Atrogin-1 levels were observed in all muscles. In addition, the activity of mTORC1 signaling was not significantly changed in aged respiratory muscles, whereas levels of pS2448-mTOR, pS65-4EBP1 (Figure 1A,C), and pS757-ULK1 (Figure 4A,B) in the gastrocnemii were significantly decreased (Table 1). Whether these changes in molecular signaling affect protein synthesis requires future investigation.

Akt mediates key cellular processes of COPD pathogenesis [42]. Components of cigarette smoke and respiratory pathogens activate Akt through cell surface receptors on alveolar macrophages and the bronchial epithelium. Consistent with Akt activation in human COPD, increases in Akt phosphorylation has been observed in the lungs of rat models of COPD and acute exacerbation of chronic obstructive pulmonary disease (AECOPD) [43,44,45]. However, cigarette smoke extract has also been reported to induce Akt degradation via TTC3, a ubiquitin ligase, in human lung fibroblasts [46], suggesting that Akt levels and activity are tightly regulated in COPD. In addition, forelimb grip strength slowly increases in COPD model rats, but the skeletal muscle contractility decreased in vitro [47]. It would be interesting to investigate which molecular signals by COPD inducers are responsible for skeletal muscle function and whether COPD inducers affect the mass and function of respiratory muscles.

Our current comparative analysis between the respiratory muscles and the gastrocnemii presents some limitations. Firstly, the current study mostly investigated molecular signaling signatures and expression levels. Although histological assessment is important for diagnosing sarcopenia and focusing on the change in the muscle mass, it was not necessary for the present study, as we primarily focused on the comparison of the molecular signaling signatures between the respiratory muscles and the gastrocnemius. This approach was appropriate to reveal that the autophagic flux was dampened in the aged gastrocnemius muscles, and not in the intercostal muscles or diaphragm, suggesting that there is a different regulatory mechanism between the respiratory muscles and the gastrocnemius muscle. Moreover, the assessment of differences in muscle strength would be more appropriate to prove the phenotypic changes in the muscles. It has previously been reported that the decrease in muscle strength with age is much more profound than the loss of muscle mass and it does not prevent the maintenance and gain of muscle mass in older adults [6]. We found that a defect in autophagy occurred, and the expression of mitochondrial quality control-related genes decreased in aged gastrocnemii, but not in aged respiratory muscles. Autophagy is required for dispersing the damaged proteins/organelles to aid in the maintenance of smooth muscle function [1]. In addition, mitochondrial damage due to a failure in mitochondrial quality control makes the muscles susceptible to dysfunction and degeneration [48]. For this reason, we speculated that the changes in aged muscles could be more closely related to the difference in muscle strength of the aged muscles. However, the currently available in situ muscle force measurement is not applicable for respiratory muscles, since it has to be performed in an ex vivo condition. Future advances in muscle force measurement of respiratory muscles are required to better understand and corroborate the molecular changes in the muscles. Second, the main goal of the current study was to emphasize the characteristic differences in terms of the main atrophy-related signals between the respiratory muscles and gastrocnemius muscles. For this reason, we focused on selective signals, important for maintaining muscle mass, such as the Akt/mTOR pathway and muscle atrophy-related signals (ubiquitin-related protein degradation and autophagy). Thus, our approach was sufficient to address whether primary molecular signaling signatures in the respiratory muscles are different from those in the gastrocnemius muscles. Further investigation would warrant unbiased profiling of mRNA and proteins in muscles, which would be suitable for exploring the entirety of the aging-related molecular changes.

## 4. Materials and Methods

### 4.1. Antibodies, Other Reagents, and Tissues

Primary antibodies were obtained as follows: LC3B, tubulin, and MuRF1 antibodies were acquired from Novus Biologicals (Centennial, CO, USA), Abcam (Cambridge, UK), and Santa Cruz Biotechnology (Dallas, TX, USA), respectively; mTOR, pS2448-mTOR, Akt, pS473-Akt, ribosomal S6, pS235/236-ribosomal S6, 4EBP1, pS65-4EBP1, FoxO1, pS256-Foxo1, Atrogin-1, ULK1, pS757-ULK1, AMPK, and pT172-AMPK were obtained from Cell Signaling Technology (Danvers, MA, USA). All secondary antibodies were procured from Jackson Immuno Research Laboratories, Inc. (West Grove, PA, USA). The intercostal, diaphragm, and gastrocnemius muscles from male Sprague Dawley rats, either 6-month-old or 20-month-old were obtained from the Aging Tissue Bank (Pusan University, Pusan, Korea) [49].

### 4.2. Tissue Lysis, Immunoprecipitation, and Western Blot Analysis

Total protein was extracted using T-PER tissue protein extraction reagent supplied along with protease and phosphatase inhibitors (Thermo Scientific, Waltham, MA, USA). Each muscle (30 mg) was lysed in extraction buffer using steel beads and a Tissue Lyser II (Qiagen, Hilden, Germany) instrument set at 30 Hz for 1–2 min. The lysates were centrifuged at 13,000× *g* for 10 min at 4 °C to remove debris. The obtained supernatant was mixed with sodium dodecyl sulfate (SDS) sample buffer and boiled for 5 min. Proteins were subjected to electrophoresis on SDS-polyacrylamide gels and then transferred to polyvinylidene fluoride membranes (Millipore, Billerica, MA, USA). The incubation of antibodies was performed in accordance with the manufacturer’s instructions. Detection of horseradish peroxidase-conjugated secondary antibodies was conducted using Immobilon Western Chemiluminescent HRP Substrate (Millipore, Billerica, MA, USA).

### 4.3. RNA Isolation and Real-time Quantitative Reverse Transcription- Polymerase Chain Reaction (RT-qPCR)

Each muscle (30 mg) was lysed in TRIzol reagent (Thermo Fisher Scientific, Waltham, MA, USA), and the total RNA was isolated in accordance with the manufacturer’s protocol. One microgram of RNA was used to synthesize cDNA using the TOPscript^TM^ RT DryMIX kit (dT18 plus; Enzynomics, Daejeon, Korea). Real-time quantitative PCR evaluation was conducted using TOPreal^TM^ qPCR 2X PreMIX (SYBR Green with high ROX; Enzynomics, Daejeon, Korea) and a CFX384 C1000 Thermal Cycler (Bio-Rad, Hercules, CA, USA). Gene expression was normalized to mouse glyceraldehyde 3-phosphate dehydrogenase (*Gapdh*) and *18S rRNA*. All primers used for RT-qPCR are shown in Table 2.

### 4.4. Statistical Analysis

The data are presented as mean ± standard deviation (SD). Statistical significance was determined using Student’s t-test. *p* values <0.05 were considered statistically significant.

## Figures and Tables

**Table 1:** Differential changes of various signaling signatures in the aged respiratory and gastrocnemius muscles.

Molecular Signalings	Protein	Intercostal/Diaphragm	Gastrocnemius
mTORC1/Akt/FoxO1	mTOR	-/-	-
	pS2448-mTOR	-/decrease	decrease
	pS235/236-rpS6	-/-	-
	pS65-4EBP1	-/-	decrease
	pS473-Akt	-/increase	-
	pS256-FoxO1	-/increase	-
Autophagy initiation	pS757-ULK1	-/-	decrease
	pT172-AMPK	-/increase	decrease
Ubiquitin-related proteolysis	*Klf15*	-/increase	-
	MuRF1	-/-	-
	Atrogin-1	-/-	-
Autophagic flux	P62	-/-	increase
	LC3BII	-/-	increase
Mitochondrial quality control	*Pgc1α*	-/-	decrease
	*CoxII*	-/-	decrease
	*Drp1*	-/-	-
	*Pink1*	-/-	-

-: no change or changes (*p* > 0.05), increase: increase (*p* < 0.05), decrease: decrease (*p* < 0.05).

**Table 2:** Primers used for RT-qPCR analyses.

Primer Symbol	Sequence of Primer (5ʹ–3ʹ)
*rGapdh_*F	GACATGCCGCCTGGAGAAAC
*rGapdh_*R	AGCCCAGGATGCCCTTTAGT
*rKlf15_*F	CTGCAGCAAGATGTACACCAA
*rKlf15_*R	TCATCTGAGCGTGAAAACCTC
*rDrp1_*F	AAGAAAAGAAGCGGCTGACA
*rDrp1_*R	GCCAGTCTTCACACAAGCAA
*rPink1*_F	AGCGAAGCCATCTTAAGCAA
r*Pink1*_R	GCCTCGGTGACAGCTAAGTC
*rPgc1α*_F	ATGTGTCGCCTTCTTGCTCT
*rPgc1α*_R	ATCTACTGCCTGGGGACCTT
*rCoxII_F*	CGCCACATCACCAATCATAG
*rCoxII_R*	GAATTCGTAGGGAGGGAAGG
*r18S rRNA*_F	AAACGGCTACCACATCCAAG
*r18S rRNA*_R	CCTCCAATGGATCCTCGTTA

## Supplementary Materials

Supplementary Materials can be found at https://www.mdpi.com/1422-0067/21/8/2862/s1.

## Abbreviations

AMPK	AMP-activated protein kinase
Dia	diaphragm
4EBP1	the eukaryotic translation initiation factor 4E (eIF4E)-binding protein 1
FoxO	Forkhead box protein O
KLFs	Kruppel-like, zinc finger transcription factors
Gas	gastrocnemius
Int	intercostal
IRS-1	insulin receptor substrate-1
mTOR	mammalian target of rapamycin
mTORC1	mTOR complex1
ULK1	Unc-51 like autophagy activating kinase 1
