# Supplementary material for: Comparative Analyses of mTOR/Akt and Muscle Atrophy-Related Signaling in Aged Respiratory and Gastrocnemius Muscles
Source: Int J Mol Sci. 2020 Apr 20;21(8):2862. doi: 10.3390/ijms21082862 (PMC7215274; doi:10.3390/ijms21082862)
Supplement: Supplementary file 1 [file ijms-21-02862-s001.pdf]

Article

Comparative analyses of mTOR/Akt and muscle atrophy-related signaling in aged respiratory and gastrocnemius muscles

Kun Woo Kim [1,†], Hye-Jeong Cho [2,3,4,†], Sana Abdul Khaliq [2,3,4,†], Kuk Hui Son [1,*], and Mee-Sup Yoon [2,3,4,*]

[1] Department of Thoracic and Cardiovascular Surgery, Gachon University Gil Medical Center, College of Medicine, Gachon University, Incheon 21565, Republic of Korea
[2] Department of Molecular Medicine, School of Medicine
[3] Lee Gil Ya Cancer and Diabetes Institute
[4] Department of Health Sciences and Technology, GAIHST, Gachon University, Incheon 21999, Republic of Korea

† These authors contributed equally to this work
* Correspondence: dr632@gilhospital.com (K.H.S); Tel.: 82-32-460-3666 (K.H.S); msyoon@gachon.ac.kr (M.-S.Y.); Tel.:82-32-899-6067 (M.-S.Y.)

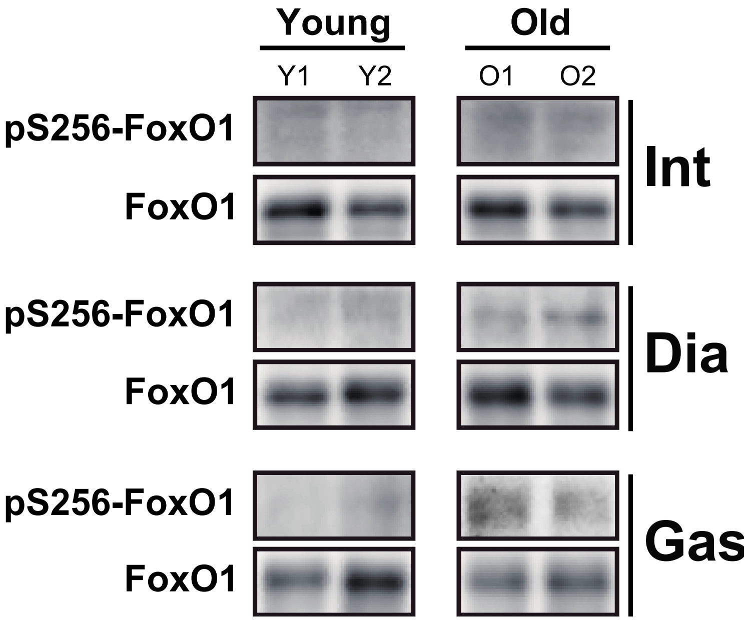

Supplementary Figure S1. Comparison of the phosphorylation of FoxO1 between the respiratory muscles and gastrocnemii with age. Each muscle was lysed and analyzed by western blotting (n=6). Young : 6-month-old rat, Old : 20-month-old rat. Abbreviations: diaphragm muscle (Dia); gastrocnemius muscle (Gas); intercostal muscle (Int).

Supplementary Table S1. Ages of Rats for Age-related studies

Title of the study	Rat strain	Ages of Young subjects (months)	Ages of Old subjects (months)	Ref
Effect of Resistance Training on Extracellular Matrix Adaptations in Skeletal Muscle of Older Rats	Wistar rats	3	21	1
Skeletal Muscle Regeneration in Very Old Rats	Wistar rats	4	32	2
Myosin heavy chain composition in young and old rat skeletal muscle	Fischer 344 rats	3	22	3
Muscle Wasting in Aged, Sarcopenic Rats Is Associated with Enhanced Activity of the Ubiquitin Proteasome Pathway	Harlan Sprague-Dawley	4	30	4
Fiber Type Composition of Unoperated Rat Soleus and Extensor Digitorum Longus Muscles after Unilateral Isotransplantation of a Foreign Muscle in Long-Term Experiments	Rats of the Lewis strain	4	17	5
Skeletal muscle metabolism in rats with low and high intrinsic aerobic capacity	N.A.	9	18	6
Ageing prolongs inflammatory marker expression in regenerating rat skeletal muscles after injury	Fischer 344 rats	3	24	7
Age-Related Gene Expression Signature in Rats Demonstrate Early, Late, and Linear Transcriptional Changes from Multiple Tissues	Sprague Dawley	6	21-27	8
Mitochondrial and Metabolic Gene Expression in the Aged Rat Heart	Male Fischer 344 x Brown Norway hybrid rats (FBN)	6	34	9
Aging-associated dysfunction of Akt/protein kinase B: S-nitrosylation and acetaminophen intervention	F344BN rats	6	27, 33	10

References

1. Guzzoni, V. *et al.* Effect of Resistance Training on Extracellular Matrix Adaptations in Skeletal Muscle of Older Rats. *Front Physiol* **9**, 374 (2018).
2. Carlson, B.M., Dedkov, E.I., Borisov, A.B. & Faulkner, J.A. Skeletal muscle regeneration in very old rats. *J Gerontol A Biol Sci Med Sci* **56**, B224-233 (2001).
3. Sullivan, V.K. *et al.* Myosin heavy chain composition in young and old rat skeletal muscle: effects of endurance exercise. *Journal of applied physiology (Bethesda, Md. : 1985)* **78**, 2115-2120 (1995).
4. Altun, M. *et al.* Muscle wasting in aged, sarcopenic rats is associated with enhanced activity of the ubiquitin proteasome pathway. *The Journal of biological chemistry* **285**, 39597-39608 (2010).
5. Soukup, T., Smerdu, V. & Zacharova, G. Fiber type composition of unoperated rat soleus and extensor digitorum longus muscles after unilateral isotransplantation of a foreign muscle in long-term experiments. *Physiol Res* **58**, 253-262 (2009).
6. Shi, M. *et al.* Skeletal muscle metabolism in rats with low and high intrinsic aerobic capacity: Effect of aging and exercise training. *PLoS One* **13**, e0208703 (2018).
7. van der Poel, C. *et al.* Ageing prolongs inflammatory marker expression in regenerating rat skeletal muscles after injury. *J Inflamm (Lond)* **8**, 41 (2011).
8. Shavlakadze, T. *et al.* Age-Related Gene Expression Signature in Rats Demonstrate Early, Late, and Linear Transcriptional Changes from Multiple Tissues. *Cell Rep* **28**, 3263-3273 e3263 (2019).
9. Barton, G.P., Sepe, J.J., McKiernan, S.H., Aiken, J.M. & Diffee, G.M. Mitochondrial and Metabolic Gene Expression in the Aged Rat Heart. *Frontiers in Physiology* **7** (2016).
10. Wu, M. *et al.* Aging-associated dysfunction of Akt/protein kinase B: S-nitrosylation and acetaminophen intervention. *PLoS One* **4**, e6430 (2009).
